# Mitochondrial ribosome biogenesis and redox sensing

**DOI:** 10.1002/2211-5463.13844

**Published:** 2024-06-07

**Authors:** Michele Brischigliaro, Ana Sierra‐Magro, Ahram Ahn, Antoni Barrientos

**Affiliations:** ^1^ Department of Neurology University of Miami Miller School of Medicine FL USA; ^2^ Department of Biochemistry and Molecular Biology University of Miami Miller School of Medicine FL USA; ^3^ Bruce W. Carter Department of Veterans Affairs VA Medical Center Miami FL USA

**Keywords:** iron–sulfur cluster, mitochondrial disease, mitochondrial ribosome, mitochondrial translation, mitoribosome assembly, redox sensing

## Abstract

Mitoribosome biogenesis is a complex process involving RNA elements encoded in the mitochondrial genome and mitoribosomal proteins typically encoded in the nuclear genome. This process is orchestrated by extra‐ribosomal proteins, nucleus‐encoded assembly factors, which play roles across all assembly stages to coordinate ribosomal RNA processing and maturation with the sequential association of ribosomal proteins. Both biochemical studies and recent cryo‐EM structures of mammalian mitoribosomes have provided insights into their assembly process. In this article, we will briefly outline the current understanding of mammalian mitoribosome biogenesis pathways and the factors involved. Special attention is devoted to the recent identification of iron–sulfur clusters as structural components of the mitoribosome and a small subunit assembly factor, the existence of redox‐sensitive cysteines in mitoribosome proteins and assembly factors, and the role they may play as redox sensor units to regulate mitochondrial translation under stress.

## Graphical abstract

Mitoribosome assembly coordinates rRNAs folding and modification with riboprotein incorporation assisted by assembly factors like RNA helicases, GTPases, and RNA‐modifying enzymes. Biochemical and structural analyses have enhanced the pathway resolution. Additionally, recent insights integrate it with mitochondrial and cellular processes such as Fe–S cluster biogenesis and redox sensing, impacting mitoribosome assembly/stability, and mitochondrial translation under oxidative stress.

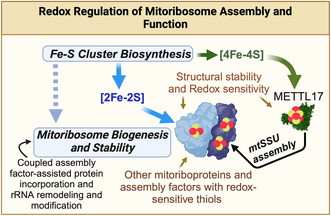

**Abbreviations**
ACO2: aconitase
BOLA3: BolA family member 3
CP: central protuberance in the mtLSU
cryo‐EM: cryogenic‐electron microscopy
ERAL1: Era G‐protein‐like 1
FASTKD2: Fas‐activated serine/threonine (FAST) kinase family protein domain
FDX2: mitochondrial ferredoxin
FDXR: ferredoxin reductase
FXN: frataxin
GEF: guanosine triphosphate exchange factor
GLRX5: glutaredoxin 5
GRSF1: G‐rich sequence binding factor 1
GTPase: guanosine triphosphatase
MCAT: malonyl‐CoA‐acyl carrier protein transacylase
METTL15: methyltransferase 15
METTL17: methyltransferase 17
MRP: mitoribosomal proteins
mtDNA: mitochondrial DNA
mtIF2: mitochondrial translation initiation factor 2
mtIF3: mitochondrial translation initiation factor 3
mtLSU: mitoribosome large subunit
mtSSU: mitoribosome small subunit
NOA1: nitric oxide associated 1 GTPase
PTC: peptidyl transferase center
RBFA: ribosome binding factor A
RCC1L^V3^
: regulator of chromatin condensation 1‐like, variant 3
ROS: reactive oxygen species
rRNA: ribosomal RNA
SILAC: stable isotope labeling by/with amino acids in cell culture
SRL: *16S rRNA* sircin‐ricin loop
TFB1M: mitochondrial transcription factor B

The synthesis of the 13 proteins encoded in the mammalian mitochondrial genome (mtDNA) occurs in dedicated mitochondrial ribosomes (mitoribosomes). These proteins are all components of the oxidative phosphorylation complexes that play essential roles in aerobic energy transduction.

The mammalian mitoribosome is a 55S ribonucleoprotein complex composed of a 39S large subunit (mtLSU) containing 52 mitoribosomal proteins (MRPs), a *16S* ribosomal RNA (rRNA), and a structural tRNA (tRNA^Val^ in human cells), along with a 28S small subunit (mtSSU) comprising 30 MRPs and a *12S rRNA*. Unlike bacterial and eukaryotic cytoplasmic ribosomes, which are approximately 60% RNA, mitochondrial ribosomes are protein‐rich, with only 25–30% RNA. While rRNAs are encoded in mitochondrial DNA (mtDNA), all MRPs are encoded in the nuclear genome, synthesized on cytoplasmic ribosomes, and imported into the mitochondrial matrix for assembly with subunit‐specific rRNAs. Mitoribosome assembly entails numerous ancillary factors, including RNA processing and modification enzymes, guanosine triphosphatases (GTPases), DEAD‐box RNA helicases, kinases, translation factors, and chaperones, which guide the maturation of mitoribosomal components and their temporal association [1, 2]. They assist in forming pre‐ribosomal particles during the assembly of individual subunits and facilitating subunit association during monosome formation [3, 4].

Comprehensive reviews on mitoribosome assembly have been published elsewhere [5, 6, 7, 8, 9]. Here, we will provide a summary of the current understanding of the process as it pertains to the mammalian mitoribosome, and the factors involved. Furthermore, we will discuss recent insights into the presence of iron–sulfur clusters and other cofactors as structural components of the mammalian mitoribosome and assembly factors, and their implications for the redox regulation of the assembly process.

## Mitoribosome structural features

**Fig. 1:**
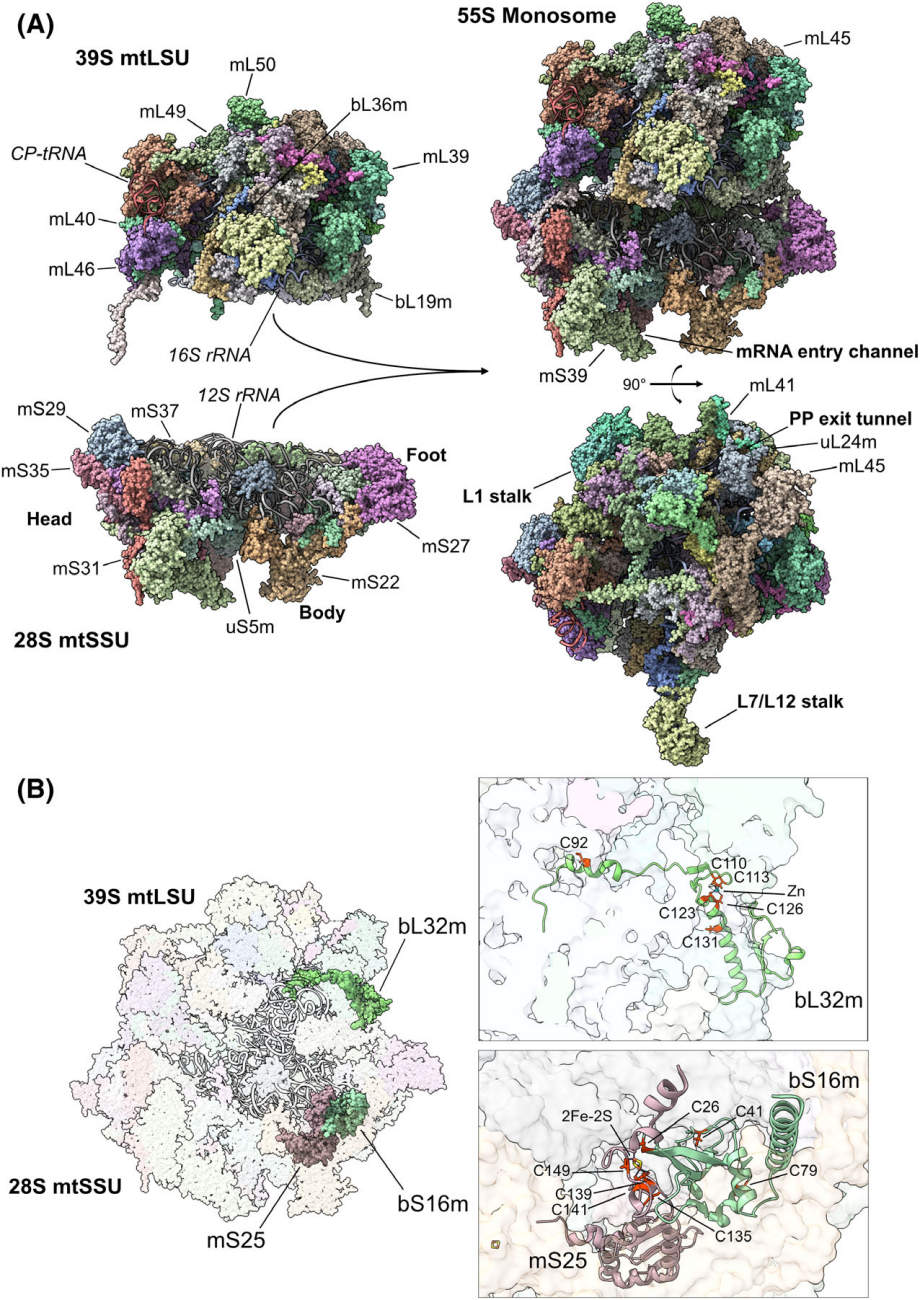
Redox sensing in the human mitochondrial ribosome. (A) High‐resolution 2.2 Å cryo‐EM structure of the human mitoribosome LSU (top left) and SSU (bottom left) and the monosome (right) (PDB ID: 7QI4). Protein components are displayed as density maps, and RNA components (*12S rRNA*, *16S rRNA*, and the central protuberance CP‐tRNA) are displayed as ribbons and labeled in italics. The main structural features of the mtSSU (head, body, foot, mRNA entry channel) and the mtLSU (polypeptide exit tunnel, L1 stalk, and L7/L12 stalk) are labeled in bold. (B) Cryo‐EM structure of the mitochondrial ribosome highlighting subunits that have redox‐responsive elements, namely mS25/bS16m and bL32m. Cysteine residues in the small subunits mS25 and bS16m coordinate an iron–sulfur cluster (2Fe–2S). Cysteine residues in the large subunit bL32m coordinate a Zinc (Zn) atom.

The application of cryo‐electron microscopy (cryo‐EM) to study human and porcine mitoribosomes has unveiled intricate details about their composition and unique features [10, 11, 12, 13] (Fig. 1). These analyses have shown that while the catalytic region at the subunit interface remains similar to that of bacterial ribosomes, mammalian mitoribosomes have undergone significant alterations. Specifically, they have lost certain ribosomal RNA (rRNA) segments and several bacterial proteins, with some conserved proteins acquiring extensions to accommodate novel positions rather than compensating for the missing rRNA [14, 15].

Moreover, mitochondrion‐specific proteins (36 out of 82 MRPs in the mammalian mitoribosome) have been observed to occupy peripheral locations within the mitoribosome structure [14] and play relevant roles during assembly (mS29, mS27, mS38 in the mtSSU, and mL37 in the mtLSU) [10, 11], tethering to the mitochondrial inner membrane (mL45) [16], and active translation, including pre‐initiation (mS37), mRNA binding (the pentatricopeptide repeat‐containing protein mS39), tRNA translocation (mL40, mL48, and mL64), and elongation (mL53) [12, 17, 18]. Interestingly, the intersubunit contacts in mammalian mitoribosomes are predominantly mediated by proteins rather than RNA, with mitochondrion‐specific bridges playing a crucial role in maintaining the structure during translation [11]. A prominent contribution is made by mS29, a GTP‐binding protein at the head of the mtSSU, which mediates 3 bridges, one of which involves a b‐hairpin supported by GDP that forms a protein:protein bridge [19].

Another notable finding is the presence of three metal‐binding motifs, initially thought to bind zinc but later identified as iron–sulfur (Fe–S) clusters. Atypically, each of these clusters is coordinated by a protein pair, two of them present in the mtSSU and one in the mtLSU [12, 20]. These clusters likely serve to stabilize the mitoribosome structure and may also play a role in sensing changes in the redox environment [12], as will be discussed later.

The unique functional features of the mammalian mitoribosome include extensive remodeling of the mtSSU mRNA channel entrance and exit compared to bacterial ribosomes [10, 11, 15, 17, 21, 22]. This remodeling is facilitated by mitochondria‐specific protein extensions and new proteins [17, 18, 22, 23]. Additionally, mS29 is crucial for intersubunit communication through a very specific mechanism. Thought to regulate translation dynamics by acting as a GTPase [5, 10, 11], recent findings suggest that this activity might not be directly linked to mitoribosome function. A recent high‐resolution cryo‐EM reconstruction of the human mitoribosome at 2.2 Å reveals an ATP coordinated by the P loop of mS29 where a GTP was previously misassigned and an additional binding site harboring a density for GDP [19]. The study concluded that GDP directly stabilizes a β‐hairpin in mS29 that acts as a molecular switch during tRNA movement and maintains the only intersubunit communication in the head region during the hybrid state [19].

Structural adaptations are also evident in the mtLSU, including modifications in the L7/L12 stalk [17], tRNA binding sites [15, 24, 25], and the presence of a structural tRNA in the place where a 5S rRNA binds in bacterial and cytoplasmic ribosomes [10, 17, 26]. Notably, the absence of certain ribosomal elements has led to the development of a unique structure termed the “P‐site finger”, formed by mL40 and mL48 [17], which compensates for missing mt‐tRNA binding sites. Furthermore, the polypeptide exit tunnel in the mitoribosome has been adapted to facilitate the transit and delivery of hydrophobic nascent polypeptides [15, 21]. The mitochondrion‐specific mL45 protein surrounds the exit tunnel and tethers the mitoribosome to the inner mitochondrial membrane [15, 21]. Membrane anchoring aligns the polypeptide delivery site with the OXA1L translocase, enabling co‐translational membrane insertion of newly synthesized proteins [27, 28].

## Mitoribosome assembly pathway and factors involved

Mitoribosome biogenesis involves a sequential pathway encompassing RNA processing, maturation, and folding for each subunit [29, 30, 31, 32]. Concurrently, mitoribosome protein sets and preassembled modules are cooperatively integrated into rRNA, forming structural clusters [3, 4, 33, 34, 35]. As in bacteria, the assembly process initiates co‐transcriptionally in mammalian mitochondria, facilitating the synchronized biogenesis of both mitoribosome subunits. In mammalian mtDNA, the rRNA genes are located on the heavy (H) strand, flanked by tRNAs in a sequence mt‐tRNA^Phe^/mtSSU rRNA/mt‐tRNA^Val^/mtLSU rRNA. Either tRNA^Val^ or tRNA^Phe^ has been identified as structural components of the mtLSU in mammals [26], resembling a bacterial operon in genomic arrangement [36]. Mitochondrial transcription is polycistronic, generating two transcripts spanning nearly the entire genome from a single L‐strand and a single H‐strand promoter, which are subsequently processed. Mitochondrial ribonuclease P (RNase P) and mitochondrial RNase Z (ELAC2) catalyze tRNA‐5′ cleavage and tRNA‐3′ cleavage, respectively, thereby releasing the rRNAs and most mRNAs [37]. It has been proposed that mitoribosome assembly initiates with a subset of 27 mtLSU proteins forming a subcomplex on unprocessed RNA containing the *16S rRNA*. This complex facilitates precursor RNA processing by RNase P and ELAC2, leading to the release of the *12S rRNA* and subsequent mtSSU assembly [30].

Insights from biochemical, genetic, and structural studies in mammalian and yeast cells, including cryo‐EM analyses of mitoribosomes from human HEK293T cells and *Trypanosoma brucei*, have shed light on the assembly process [3, 4, 34, 35, 38]. Comparative studies of the mammalian mitoribosome assembly line with bacterial and yeast ribosomes have been published elsewhere [5, 9]. Human mitoribosome biogenesis requires trans‐acting factors, such as RNA modifying enzymes, GTPases, RNA helicases, and phosphatases, acting at specific steps of the process [5].

Various methodologies have been employed to enhance our comprehension of the mitoribosome assembly pathway in mammalian cells. Stable isotope labeling with amino acids in cell culture (SILAC) pulse‐labeling in HeLa cells revealed the rates of MRP mitochondrial import and assembly into 55S mitoribosomes, offering a useful – albeit low‐resolution –assembly model [4]. Human mitoribosome assembly takes approximately 2–3 h, slower than bacterial ribosome assembly, possibly due to the nuclear genetic origin of MRPs, which are synthesized in excess, imported into mitochondria, and regulated by degradation of unassembled protein fractions [4]. To identify potential mitoribosome assembly factors, researchers have screened for mitochondrial RNA binding proteins [39, 40], while others have conducted proteomics analyses of the mitoribosome interactome [2, 29, 41, 42]. Additionally, some groups have leveraged the well‐understood bacterial ribosome assembly pathway [43, 44] and screens in *Saccharomyces cerevisiae* [1, 45, 46] to uncover conserved factors in mammals. These identified assembly factors have subsequently been characterized in human cultured cells or mouse models [30, 47, 48]. Furthermore, investigations into patients with mitochondrial translation deficiency disorders have revealed novel assembly factors and underscored the significance of MRPs in mitoribosome assembly across various tissues [49, 50, 51, 52]. Finally, cryo‐EM investigations have shed light on the temporal aspects of rRNA folding and protein integration during the final stages of ribosomal maturation, particularly when stable assembly intermediates accumulate sufficiently for characterization. To date, only one study has reported structures of late‐stage assembly intermediates of the human mtLSU, isolated from a native pool within a non‐modified wild‐type human cell line (HEK293T) [38]. Other studies have utilized genetic perturbations in wild‐type human cells to enrich mitoribosome subassemblies, either of the mtSSU [12] or mtLSU [53, 54, 55, 56, 57, 58]. The findings from these studies have been synthesized into a stepwise model of mtLSU biogenesis [9], which will be summarized in the following section.

### Assembly pathway for the mtSSU

**Fig. 2:**
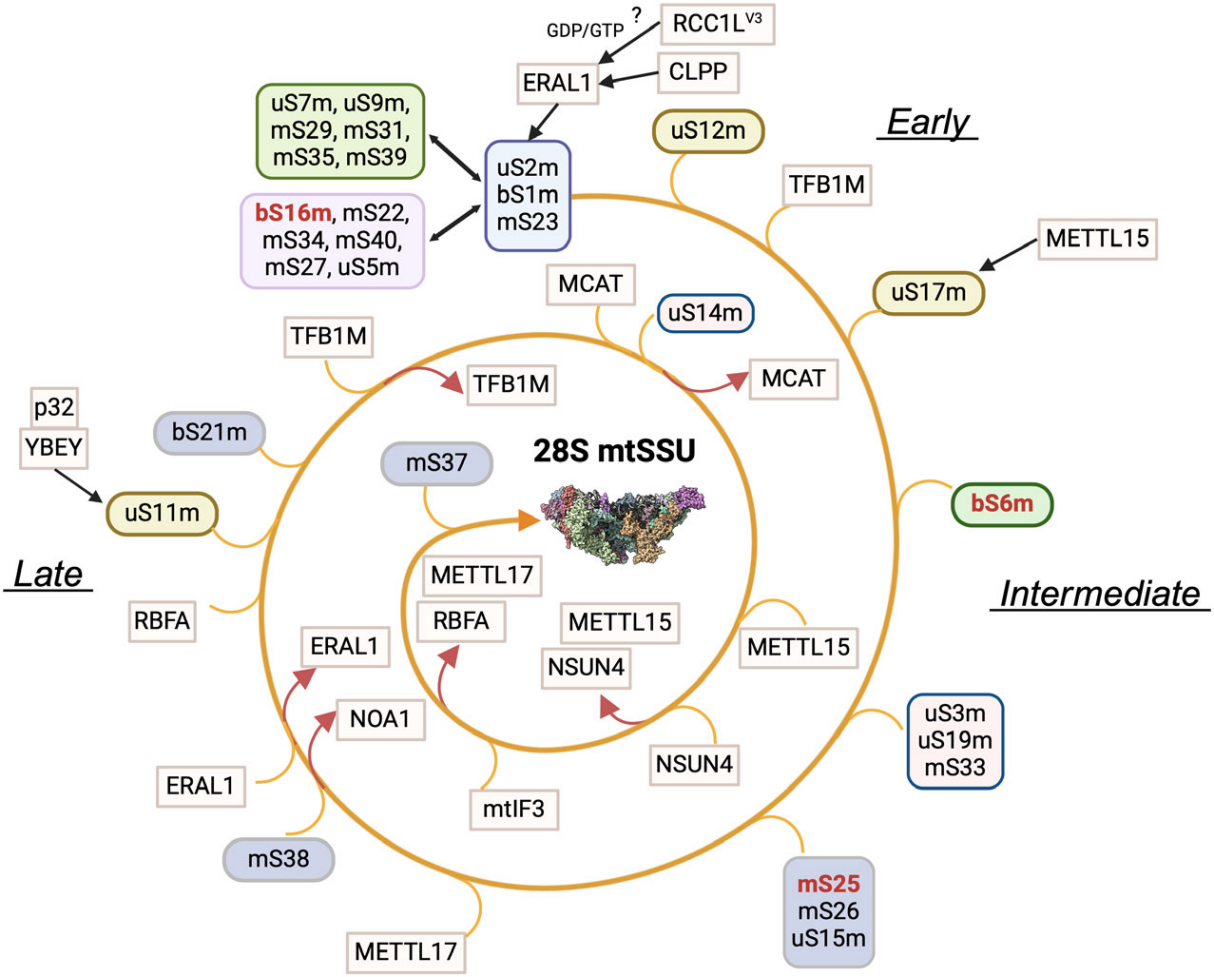
Mitoribosome SSU assembly. Model for the assembly pathway for the human 28S mt‐SSU. delineating the recruitment of proteins and the incorporation and release of known assembly factors in their respective stages of assembly. Individual proteins and protein clusters are depicted using distinct boxes. Proteins involved in [Fe–S] cluster coordination are marked in red. The rectangular boxes highlight assembly factors essential for mitoribosome biogenesis. The figures were prepared using biorender and chimerax [126]. The maturation of the *12S rRNA* is not depicted. ? indicates proposed but not experimentally proven steps during the assembly process.

The current model of mtSSU biogenesis is outlined in Fig. 2.

The SILAC proteomics study revealed that mtSSU protein assembly begins with the incorporation of two large protein modules [4]. One module attaches to the lower body/foot of the mtSSU and contacts the 5′ and 3′ *12S rRNA* domains, involving uS5m, bS16m, mS22, mS27, mS34, and mS40, while the other module localizes to the head region, including uS7m, uS9m, mS29, mS31, mS35, and mS39 and extends through the major 3′ domain in the *12S rRNA* [4]. Additional proteins such as mS23, uS2m, and bS1m interact with both modules. Further, three proteins (uS11m, uS17m, and uS12m) bind independently to the mtSSU outer surface and are proposed to be essential for the recruitment of the late assembly proteins [4]. However, structural studies have shown that uS11m gets incorporated during the intermediate/late assembly stages [20].

Several assembly factors facilitate early mtSSU biogenesis. The GTPase ERAL1 (Era G‐protein‐like 1) acts as an RNA chaperone to stabilize *12S rRNA* [47], while TFB1M (mitochondrial transcription factor B) catalyzes subsequent methylation [59]. TFB1M remains bound to the maturing mtSSU until the late stages of assembly [12]. The ATP‐dependent protease CLPP regulates ERAL1 levels [60]. The GTPase MTG3, the putative GDP/GTP exchange factor RCC1L^V3^ (regulator of chromatin condensation 1‐like, variant 3), and the G‐rich sequence binding factor 1 (GRSF1) are also involved in early‐mid stages of mtSSU assembly, although their precise functions are not fully understood (reviewed in Ref. [61]).

Most late‐binding proteins localize to the interface with the mtLSU and include some that incorporate as single units (e.g., mS38) and two clusters [4]. The cluster uS14m‐uS10m‐uS3m‐mS33 binds in the head region in association with the early uS7m‐mS29 group, and the cluster uS15m‐mS25‐mS26 binds near the early bS16m‐mS22 group [4].

Cryo‐EM studies have elucidated the mid‐late, and final steps of mtSSU biogenesis, revealing the regulatory role played by several assembly and maturation factors [12, 20]. To capture human mtSSU assembly intermediates, researchers used several strategies. In a comprehensive study, the authors engineered a cell line expressing a functional tagged version of the putative methyltransferase METTL17 and used affinity purification approaches [20]. Six METTL17‐bound mtSSU assembly intermediates were characterized, progressing based on the proportion of unfolded rRNA and the presence of assembly factors and mtSSU proteins [20]. They revealed the structures and functions of six assembly factors in the context of assembly: METTL17, the GTPases NOA1 [62] and ERAL1 [47], the methyltransferase TFB1M [63], the rRNA chaperone RBFA [64], and MCAT (malonyl‐CoA‐acyl carrier protein transacylase), a protein that also functions in mitochondrial fatty acid metabolism [65]. Despite their ordering in a linear pathway, the authors stressed the possibility of alternative pathways, including those in which certain proteins undergo multiple cycles of binding and dissociation [20]. Notably, the data indicates a decoupling of head and body assembly mechanisms during maturation, suggesting that mtSSU assembly is not strictly linear. The least mature mtSSU intermediate identified lacks mS38, uS11m, bS21m, and mS37, and also exhibits an immature decoding center and platform. The assembly factors previously mentioned facilitate the maturation of the decoding center, compacting the platform and stabilizing the 3′ end rRNA, thereby enabling the timely methylation of crucial residues in *12S rRNA* helix 45 (h45) during assembly.

Three major mtSSU assembly steps were identified [20]. First, the maturation of the decoding center starts with the stabilization of the platform and 3′ end rRNA and involves binding of METTL17, stabilization of *12S rRNA* h44 and h45, incorporation of mS38, and release of NOA1, followed by the binding and release of ERAL1, and the incorporation of RBFA with uS11m and bS21m. It was previously shown that the endoribonuclease YBEY interacts with the multifunctional protein p32 and aids in uS11m incorporation [66]. Second, the methylation of h45, which involves the binding and release of TFB1M and remodeling of h23, followed by the binding of MCAT to promote maturation of the head rRNA and decoding center. Third, the maturation process continues with the incorporation of uS14m, the release of MCAT, further maturation of the head rRNA and decoding center, and the release of METTL17 and RBFA.

In another work [12], the authors used a cell line depleted of TRMT2B, the enzyme responsible for the formation of 5‐methyluridine [67, 68], and characterized four accumulating late‐stage mtSSU intermediates that involve RBFA [64] and the methyltransferases TFB1M and METTL15 [69, 70], together with the protein mS37 and the initiation factors mtIF2 and mtIF3. In this study, the most immature particle contains all the MRPs except mS37, has partially disordered rRNA helices (h44 and h45), and contains the assembly factors RBFA and TFB1M, which maintains a partially disordered mS38. The final steps involve the release of TFB1M, final maturation of mS38, and further *12S rRNA* maturation [12], which leads to the recruitment of METTL15 by RBFA, possibly independently of direct rRNA binding [69]. This promotes further rRNA maturation and partially displaces RBFA, positioning it to block mRNA docking to prevent premature mRNA recruitment. At this point, all known rRNA modifications are already present [12], suggesting that METTL15 might facilitate the recruitment of the m^5^C methyltransferase NSUN4 for late rRNA modification [5].

The new RBFA conformation allows the initiation factor mtIF3 to replace METTL15 and already occupy the subunit interface during the assembly. Finally, the mitochondria‐specific ribosomal protein mS37 outcompetes RBFA to complete the assembly with the mtSSU–mS37–mtIF3 complex [71], which proceeds toward mtIF2 binding and translation initiation [12].

### Assembly pathway for the mtLSU

**Fig. 3:**
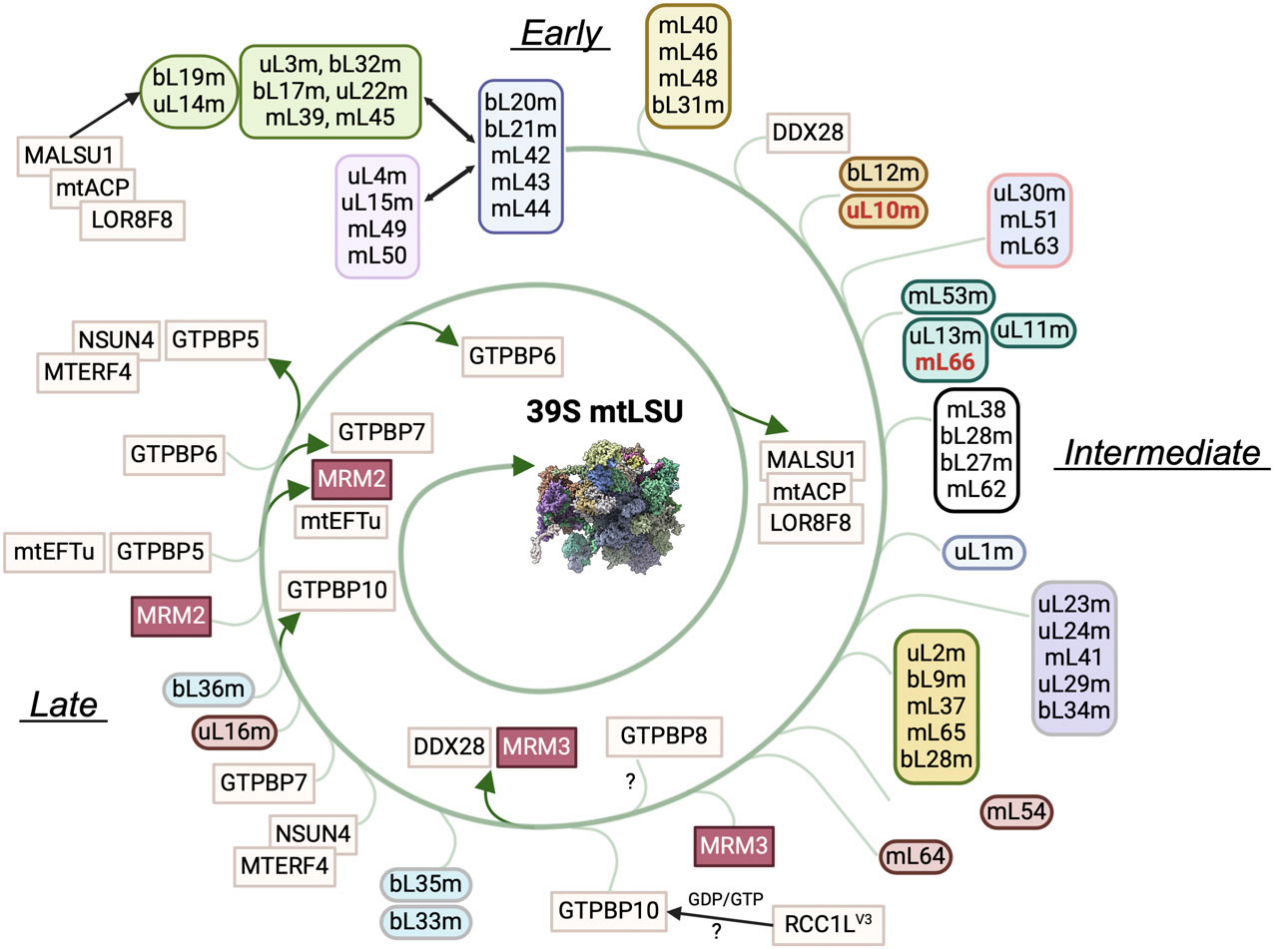
Mitoribosome LSU assembly. Model for the assembly pathway for the human 39S mt‐LSU, delineating the recruitment of proteins and the incorporation and release of known assembly factors in their respective stages of assembly. Individual proteins and protein clusters are depicted using distinct boxes. Proteins critical to iron–sulfur (Fe–S) cluster coordination are marked in red. The rectangular boxes highlight assembly factors essential for mitoribosome biogenesis. The figures were prepared using biorender and chimerax [126]. The maturation of the *16S rRNA* is not depicted. ? indicates proposed but not experimentally proven steps during the assembly process.

The current model of mtLSU assembly is delineated in Fig. 3.

The SILAC proteomics study identified three distinct stages, early, intermediate, and late, in mtLSU assembly [4]. In the early phase, coordinated binding of three large protein clusters occurs mainly within the region encompassing the 5′ rRNA domain. A first cluster, formed by rRNA binding MRPs uL3m and bL19m, and other proteins (uL14m, bL17m, uL22m, and bL32m) anchors mL39, and then mL45, which tethers the mtLSU to the inner membrane. In this module, the assembly factor MALSU1 aids uL14m insertion [72, 73], while the DEAD‐box RNA helicase DDX28 and the Fas‐activated serine/threonine (FAST) kinase family protein FASTKD2 [41, 42, 74] stabilize the *16S rRNA*. A second cluster comprises the mRNA‐binding protein bL20m along with bL21m, mL42, mL43, and mL44. A third cluster is formed by the mRNA binding heterodimer uL4m‐uL15m, recruiting mL49 and mL50. The fourth early‐assembly cluster, including proteins mL40, mL46, mL48, and bL31m, is associated with the tRNA^Val^ in the mtLSU central protuberance (CP) [4].

During the intermediate stage, the fourth early cluster facilitates the incorporation of mL38, uL18m, and bL27m at the CP, while the uL13m‐mL66 dimer and uL11m bind the uL10m stalk [4]. A subsequent step is proposed to involve the incorporation of a large module composed of MRPs mL41, uL23m, uL24m, uL29m, and bL34m, forming the polypeptide exit tunnel [4]. However, this needs confirmation because some of these MRPs assemble early in bacteria and yeast mitochondria [3, 75, 76].

Late‐stage mtLSU assembly involves the incorporation of proteins positioned at the interface with the mtSSU, including uL2m, uL28m, uL29m, mL37, and mL65, some of which form intersubunit bridges. In agreement, cryo‐EM studies reveal that the intersubunit interface attains organization only in late assembly stages [38]. Multiple assembly factors play crucial roles in finalizing mtLSU maturation and establishing quality‐control checkpoints, particularly concerning the peptidyl transferase center (PTC) formation. At this stage, at least four GTPases, including GTPBP7/MTG1, two homologs of bacterial ObgE (GTPBP5/MTG2, and GTPBP10), and GTPBP6, are involved, orchestrating sequential recruitment as suggested by proteomics, biochemical, and structural studies (reviewed in Ref. [6]) (Fig. 3).

All human mtLSU intermediates analyzed by cryo‐EM are late‐stage. The earliest lacks bL33m, bL35m, and bL36m, displaying an immature CP and delocalized *16S rRNA* domains IV and V. It is bound by assembly factors DDX28, GTPBP10, the 2′‐O‐methyltransferase MRM3, and the MALSU1‐mtACP‐LOR8F8 module [38, 54, 72]. The MALSU1‐containing module remains bound to uL14m, bL19m, and the *16S rRNA* sarcin‐ricin loop (SRL, h95) until mtLSU assembly is finalized [38], likely obstructing intersubunit bridge B8 formation and preventing premature subunit joining. DDX28 binds early to *16S rRNA* h88 and persists until late maturation stages [42], stabilizing the CP in an immature conformation. GTPBP10 interacts with the SRL and L12 stalk base, facilitating MRM3 action on *16S rRNA* domain V [54]. Loss of GTPBP10 in a KO cell line prevents incorporation of specific proteins (bL33m, bL34m, bL35m, and bL36m [2]), consistent with cryo‐EM findings. GTPBP10 may be regulated by the GTP/GDP exchange factor RCC1L^V1^ [77]. Interestingly, a deacylated tRNA was found in the ribosomal E‐site in this pre‐mtLSU maturation intermediate, suggesting a role in stabilizing intermediates or monitoring CP folding state before completion [54]. Of the two human ObgE‐homolog proteins, GTPBP10 acts prior to GTPBP5 during mtLSU biogenesis [78]. The order of action is deduced from the fact that while the GTPBP10‐bound state is characterized by a large displacement of h89 on *16S rRNA* domain V, h89 has already adopted a fully mature conformation in a GTPBP5‐bound mtLSU intermediate [78]. Therefore, the two mitochondrial homologs of ObgE, required for bacterial LSU h89 folding and incorporation of L16 and L36, perform complementary but distinct functions in mitoribosome *16S rRNA* h89 folding [78], explaining why the two isoforms exist in human mitochondria.

Biochemical studies indicate that the next recruited assembly factor is likely GTPBP7, which interacts with domain VI helices in the *16S rRNA* and bL19m. This interaction induces a conformational change in the bL19m‐containing domain, facilitating the incorporation of late assembly proteins bL36m and bL35m, thereby completing mtLSU formation [79]. Cryo‐EM captured GTPBP7 in an assembly intermediate only in the presence of GMPPCP, where it is locked in a pre‐hydrolysis conformation, bound to the MTERF4‐NSUN4 complex [57]. Here, GTPBP7 interacts with h92 and directly contacts U3039 in the *16S rRNA* A loop to monitor MRM2‐dependent methylation status. Upon MRM2 recruitment, GTPBP7 undergoes a conformational change, suggesting its ability to bind different mtLSU locations in distinct states [55, 57]. GTPBP7 remains bound to the mtLSU until maturation is completed, and prior to subunit joining. GTPBP7 has been proposed to interact with mtSSU protein mS27, potentially functioning as a guanosine triphosphate exchange factor (GEF) to promote its release from the ribosome, enabling the formation of the mB6 intersubunit bridge between bL19m and mS27 [79]. Subsequent recruitment of GTPBP5 induces an assembly state in which the *16S rRNA* A loop is displaced from the active site of MRM2 [55, 80], the methyltransferase that catalyzes the 2′‐O‐methyl modification at position U1369, an essential PTC component [32, 81]. Cryo‐EM captured a particle containing GTPBP7 and GTPBP10, which cooperate in the folding of the *16S mRNA* domain V [78], and another with GTPBP7, GTPBP5, and MRM2, suggesting simultaneous action [57].

All these structural studies have revealed that the final stages of mtLSU assembly primarily focus on remodeling interfacial RNA elements within nearly fully formed particles. However, proteins of the CP, uL16m, and bL36m are incorporated and stabilized during the final steps. In this line, cells devoid of MRM2 accumulate late assembly stage mtLSU particles containing the MALSU1‐L0R8F8‐mtACP anti‐association module, unstructured interfacial RNA components, and lacking bL36m [38, 58], as observed in a minor fraction of homeostatic assembly intermediates. However, in the absence of MRM2, most of these particles (~ 85%), lack mitochondrion‐specific CP elements (mL40, mL46, mL48) and display a poorly defined structural mt‐tRNA [58], suggesting that, in contrast to the observations made by SILAC proteomics, their incorporation is likely a very late event during mtLSU maturation. Another study identified a particle containing NSUN4‐MTERF4 and the MALSU1‐L0R8F8‐mtACP module in the presence of GTPBP7 and GTPBP10 [78]. This particle lacks uL16m and bL36m. It has been proposed that GTPBP10 guides h89 into its ribosomal cavity, facilitating proper docking of uL16m and bL36m, leading to GTP hydrolysis in GTPBP10 and its dissociation from the ribosome. GTPBP7 may persist on the mtLSU with a partially unstructured h89 base. The incorporation of uL16m could induce GTP hydrolysis, reorganizing GTPBP7 on the RNA surface to allow MRM2 binding, which modifies U3039 in the A loop.

A newly identified conserved mtLSU assembly GTPase, GTPBP8, has been characterized biochemically [82]. Its absence results in abnormal mtLSU accumulation and a significant decrease in fully assembled 55S monosomes [82]. GTPBP8 interacts with uL16m and bL31m during the late stages of mtLSU assembly, likely functioning in conjunction with the other GTPases.

After *16S rRNA* maturation by MRM2 and MRM3‐catalyzed methylation, and potentially pseudouridylation by the pseudouridine synthase RPUSD4 [29, 83], modifying enzymes would dissociate [80]. As mentioned earlier, GTPBP5 completes h89 base folding, releasing the A loop from MRM2 and potentially prompting the departure of at least MRM2 and GTPBP7 from the mtLSU intermediate [78]. GTPBP5 and MTERF4‐NSUN4 remain bound in this intermediate, where NSUN4 and GTPBP5 cooperate to fold the PTC [53, 56]. Subsequent release of MTERF4‐NSUN4 and GTPBP5 leads to GTPBP6 recruitment, akin to the bacterial ribosome splitting factor HflX, further aiding PTC folding [53, 84]. The release of GTPBP6 results in a final intermediate where all elements are present, with the MALSU1‐mtACP‐LOR8F8 module still bound [38], acting as the last anti‐association complex preventing premature subunit joining.

## Cysteine‐containing mitoribosome proteins and assembly factors and redox control of mitoribosome biogenesis

### Redox control of mitochondrial translation

The rate of global translation and, specifically, mitochondrial protein synthesis are reversibly decreased upon exposure to exogenous and intracellular mitochondria‐originated oxidative stress [85]. These observations support an often‐overlooked theory proposed by Allen 30 years ago: that organelle gene expression, especially those involved in electron transport and energy coupling, is tightly regulated by redox potentials [86]. This regulation is expected to be crucial to mitigating the harmful effects of occasional disruptions in electron pathways and can be particularly relevant in the context of electron transport disturbances underlying mitochondrial disorders. Mitochondria originated some 2 billion years ago through a fateful endosymbiosis between a prokaryote capable of aerobic energy transduction and a protoeukaryote [87]. During evolution, most genes of the endosymbiont were transferred to the nucleus of the host cell, but a few remain in organellar genomes. These genes code for ribosomal and transfer RNAs relevant for mitochondrial translation, as well as core subunits of the OXPHOS system. While it could simply be a matter of evolutionary time, several hypotheses have been proposed to explain why mitochondrial DNA persists [88, 89]. Among them, it has been suggested that organelle genomes have been retained because they are necessary for synthesizing structural proteins of the respiratory chain within bioenergetic membranes, crucial for maintaining redox balance and safeguarding the cell against deadly consequences of electron transport disruptions [88].

Current studies are examining the mechanisms by which the mitochondrial translation machinery senses oxidative stress to attenuate protein synthesis. The recent identification of redox‐sensitive iron–sulfur clusters [90, 91] and thiols [85, 92] in mitoribosome proteins and assembly factors suggests mechanisms by which reversible changes in translation machinery occur by adjusting the redox state of translation‐related proteins, thereby controlling protein synthesis. These mechanisms will be discussed in this section.

### Mitoribosomes, iron–sulfur clusters, and redox sensing

Iron–sulfur clusters are represented by three common configurations, [2Fe–2S], [3Fe–4S], and [4Fe–4S], although they can also exist in more complex arrays such as the [8Fe–7S] center seen in nitrogenases [93]. Typically, these clusters feature iron ions interconnected by inorganic sulfide and bound to proteins via cysteine residues, though occasionally other ligands like histidine, arginine, lysine, serine, and water are involved [94, 95, 96, 97]. The ability of the clusters to toggle between oxidation states of +2 and +3 allows for sophisticated redox chemistry, with potentials spanning from −500 to 300 mV [98]. In eukaryotes, iron–sulfur clusters localize within various cellular compartments such as the mitochondrion, cytosol, endoplasmic reticulum, and nucleus. Functionally, iron–sulfur clusters play pivotal roles in both redox reactions and a plethora of non‐redox processes, ranging from mitochondrial respiration to enzymatic reactions, gene regulation, DNA/RNA metabolism, structural support, and detecting oxygen radicals and other molecules [99, 100, 101, 102, 103, 104, 105, 106, 107]. Recent findings revealing the presence of three [2Fe–2S] clusters in the mammalian mitoribosome and one [4Fe–4S] cluster in a mtSSU assembly factor have connected [Fe–S] biosynthesis and delivery to mitoribosome biogenesis.

A 2.21 Å resolution cryo‐EM map of the human mitoribosome allowed the detection of three [2Fe–2S] clusters, each coordinated by a pair of mitoribosome proteins, each providing three or one cysteine thiolates [12]. Within the mtLSU, one [2Fe–2S] cluster is located at the L7/L12 stalk base, bridging mitochondrion‐specific mL66 and the N‐terminal extension of uL10m. In the mtSSU, one [2Fe–2S] cluster connects mitochondrion‐specific extensions of bS18m with three coordinating cysteines and bS6m with one, while another cluster located to the lower body is coordinated by three cysteines in mS25 and a fourth one in bS16m [12] (Fig. 1B). Despite their peripheral location [33], these clusters are vital for mitoribosome function, and mutations in some of the coordinating proteins have been associated with human mitochondrial disorders. Mutations in mS25 lead to encephalomyopathy [108], while mutations in its 2Fe–2S‐coordinating partner bS16m result in a syndrome characterized by corpus callosum agenesis, hypotonia, and fatal neonatal lactic acidosis [109]. Structure–function correlation studies based on mutagenesis of [2Fe–2S] cluster‐coordinating cysteines have unraveled the role of the clusters in standard growth conditions in the structural stabilization of the involved mitoribosome proteins and subunits [90].

Inducing the loss of the Fe–S cluster biosynthesis protein frataxin (FXN) via CRISPR in human K562 cells led to the anticipated depletion of Fe–S cluster‐containing proteins. However, it also caused a reduction in mitochondrial protein synthesis [110]. This translation impairment was linked to the absence of the conserved methyltransferase‐like protein METTL17, which associates with the *12S rRNA* in the mtSSU head during the late assembly stages to promote the rearrangement of several helices and block the mRNA channel to prevent mRNA premature binding [110]. Notably, METTL17 was found to contain a previously unidentified [4Fe–4S] cluster essential for its binding to the mitoribosome [110].

In mammalian cells, the biogenesis and distribution of Fe–S clusters involve two distinct machineries located in the mitochondria and the cytosol. Excellent reviews on the topic can be found elsewhere [111, 112, 113, 114]. Briefly, *de novo* Fe–S cluster biosynthesis initiates in mitochondria with the assembly of a [2Fe–2S] cluster on a complex comprising the scaffold protein ISCU2, the cysteine desulfurase complex NFS1/LYRM4/ACP1, and FXN. Electron supply for cluster formation is facilitated by the mitochondrial ferredoxin (FDX2)/ferredoxin reductase (FDXR) system. Once synthesized, the cluster is transferred to target proteins either via monothiol glutaredoxin GLRX5 or through an apo GLRX5‐BOLA3 heterodimeric complex, enabling subsequent transfer to NFU1 and other unidentified targets. Formation of [4Fe–4S] clusters involves the ISCA1 and ISCA2 proteins, which couple two [2Fe–2S] clusters to generate a [4Fe–4S] cluster. These clusters are transferred directly to target apoproteins like aconitase (ACO2) or through targeting factors such as NFU1. NFU1 aids in delivering [4Fe–4S] clusters to specific apo‐ target proteins, assisted by additional factors like NUBPL for electron transport chain complex I or BOLA3 for lipoyl synthase. A systematic silencing of Fe–S biosynthesis and delivery factors, coupled with quantitative proteomic analyses, has revealed GLRX5 and BOLA3 as the mitoribosome [2Fe–2S] cluster delivery factors [90]. Additionally, ISCA1 and NFU1 have been identified as factors responsible for targeting a [4Fe–4S] cluster to the assembly factor METTL17 [90].

Certain [Fe–S] cluster proteins have been recognized for their ability to sense and regulate gene transcription and translation in response to environmental cues like oxidative stress. Given the peripheral positions of the mitoribosomal [2Fe–2S] clusters, there is a potential for involvement in oxidative stress sensing. These clusters may lose their integrity or undergo redox modifications, triggering conformational changes that modulate their activity. Thiol trapping assays demonstrated that, within the mitoribosome, at least the [2Fe–2S] cluster coordinated by mS25‐bS16m is susceptible to H_2_O_2_ [90] (Table 1), potentially contributing to the attenuation of the observed mitochondrial protein synthesis during oxidative stress [90]. Moreover, the [4Fe–4S] cluster in METTL17 exhibits high sensitivity to H_2_O_2_ [90] (Table 2), likely serving to inhibit new mtSSU assembly under oxidative stress conditions. Therefore, the mitoribosome [2Fe–2S] clusters play a role not only in the structural stabilization of the involved proteins and subunits but also in oxidative stress sensing to regulate mitochondrial translation accordingly [90].

**Table 1:** Redox‐sensitive cysteines in human mitoribosome proteins. The proteins are named according the unified nomenclature [124]. The old nomenclature is presented as a reference. Identified highly H_2_O_2_‐sensitive cysteine residues [92] are highlighted in bold. Proteins containing cysteine‐coordinating [2Fe–2S] clusters are in red, and those coordinating Zn^2+^ are in blue.

Protein name	Old nomenclature	Redox‐sensitive cysteines	Ref.
*Mitoribosome small subunit*			
uS2m	MRPS2	C162	[92]
uS5m	MRPS5	C211, C324, C377	[92]
bS6m	MRPS6	C105	[92]
uS7m	MRPS7	C152	[92]
uS9m	MRPS9	C233, C330, C353	[92]
uS11m	MRPS11	C112	[92]
uS12m	MRPS12	C132	[92]
uS17m	MRPS17	C153, C195	[92]
bS16m	MRPS16	C26	[90]
bS21m	MRPS21	**C87**	[92]
uS3m	MRPS24	C120	[92]
mS25	MRPS25	C139, C141, C149	[90, 92]
mS27	MRPS27	**C49**, C276	[92]
mS35	MRPS35	**C212**	[92]
*Mitoribosome large subunit*			
uL2m	MRPL2	C213, C240	[92]
uL3m	MRPL3	**C138**	[92]
uL10m	MRPL10	C180	[92]
uL11m	MRPL11	C50	[92]
uL18m	MRPL18	C125	[92]
bL21m	MRPL21	C124, C203	[92]
bL28m	MRPL28	C154	[92]
bL32m	MRPL32	C104, C107, C117, C120	[85, 125]
bL35m	MRPL35	C150	[92]
mL37	MRPL37	**C153**, C177, C203	[92]
mL38	MRPL38	**C252, C290**	[92]
mL39	MRPL39	C133, C146, **C335**	[92]
mL43	MRPL43	C91	[92]
mL44	MRPL44	C96, C136, C276	[92]
mL45	MRPL45	C80, C199	[92]
mL53	MRPL53	C21, C49, C63	[92]
mL65	MRPS30	C316, C393	[92]
mL66	MRPS18A	C70, C108, **C186**	[92]

**Table 2:** Redox‐sensitive cysteines in human mitoribosome assembly factors. Highly H_2_O_2_‐sensitive cysteine residues are highlighted in bold [92]. Proteins containing cysteine‐coordinating [4Fe–4S] clusters are in red.

Protein name	Protein class	Redox‐sensitive cysteines	Ref.
**Mitoribosome assembly factors**			
*12S rRNA* modification			
TFB1M	Methyltransferase	C160, C204, C304	[92]
NSUN4	Methyltransferase	C258	[92]
*mtSSU assembly*			
ERAL1	GTPase	C150	[92]
RCC1L^V3^	GEF	C181, C451	[92]
RBFA	rRNA maturation	C136	[92]
GRSF1	RNA binding	C476	[92]
*mtLSU assembly*			
DDX28	DEAD‐box RNA helicase	**C170**	[92]
DHX30	DEAD‐box RNA helicase	C118, C309, **C484**, **C606**, C673, C1183	[92]
GTPBP5	GTPase	**C206**	[92]
GTPB7	GTPase	C113, C211	[92]
GTPBP10	GTPase	C88	[92]
MALSU1	DUF143 domain containing protein	C233	[92]
METTL17	Methyltransferase‐like	**C333, C339, C347, C404**	[90]
mtEFTu	Translation elongation factor	C147, C222, **C290**	[92]
MTERF4	Transcription termination factor family	C69	[92]
FASTKD2	FAST Kinase‐domain containing protein	C377, C638, C645	[92]
RPUSD3	Pseudouridine synthase	C147, **C345**	[92]

A functional link between ribosome assembly and biogenesis of iron–sulfur proteins was previously established for cytosolic and bacterial translation systems. In *S. cerevisiae*, the protein RNase L inhibitor (Rli1), containing a [4Fe–4S] cluster, links ribosome assembly, translation, and recycling [115, 116, 117, 118, 119]. Under oxidative stress, this cluster senses reactive oxygen species (ROS), and Rli1 loses activity thereby reducing protein synthesis [120]. Similarly, in *Escherichia coli*, the enzyme RumA, also with a [4Fe–4S] cluster, methylates rRNA and senses ROS, which induce protein degradation, regulating its activity [121]. In mitochondria, H_2_O_2_ treatment is sensed by both, the [4Fe–4S] cluster in METTL17 and the mitoribosome [2Fe–2S] clusters, leading to cluster loss or remodeling and subsequent attenuation of mitoribosomal assembly and translation [90].

### Other mitoribosome proteins and assembly factors containing redox‐sensitive cysteine residues

Recent reports suggest that the attenuation of mitochondrial translation induced by H_2_O_2_ could additionally result from the modification of other redox‐sensitive cysteines, including those in ribosomal proteins and assembly factors but not involved in [Fe–S] cluster coordination [85, 92]. By using the OxICAT technology [122], which combines differential thiol trapping and isotope‐coded affinity tagging to allow for the site‐specific quantification of the fraction of protein thiol oxidation *in vivo*, it has been shown that the conserved mtLSU protein bL32m undergoes redox‐dependent regulation in yeast [85]. Yeast and human bL32m contain four cysteines that coordinate a Zn^2+^ ion, which anticipates the two proteins having similar sensitivity to oxidative insults (Fig. 1B and Table 1). Two other conserved zinc finger proteins, mS40 and bL36m, exists in the mtSSU and mtLSU, respectively, although they have not been found to undergo redox changes upon exposure to oxidative stress.

In another study, researchers applied quantitative proteomics to identify redox‐sensitive cysteine residues within human mitochondria using the isotopic tandem orthogonal proteolysis‐activity‐based protein profiling (isoTOP‐ABPP) approach in combination with a modified OxICAT technology [92]. This study revealed the presence of 19 oxidative stress‐sensitive cysteines within 13 proteins of the mtSSU, 26 within 17 mtLSU proteins, and 30 within 16 assembly factors (Tables 1 and 2). Notably, the impact of increasing H_2_O_2_ concentrations was not uniform across the identified cysteines, with some displaying heightened sensitivity, such as C87 in bS21m, C49 in mS27, C138 in uL3m, C252 in mL38, C186 in mL66, and C335 in mL39 (Table 1), as well as C170 in DDX28, C484 and C606 in DHX30, C290 in mtEF‐Tu, and C206 in GTPB5 (Table 2). None of these cysteines have been reported to coordinate any metal center or participate in disulfide bonds. Nonetheless, several of them might play crucial roles in maintaining protein structure or facilitating protein–protein interactions within the mitoribosome complex, suggesting that their oxidation could significantly impact mitochondrial ribosome biogenesis and/or activity. These observations underscore another potential mechanism through which oxidative stress may influence mitochondrial translation.

## Conclusions and perspectives

The field of mammalian mitoribosome biogenesis has seen significant growth in the past decade, driven by advancements in mass spectrometry, cryo‐electron microscopy, and gene editing techniques in human cell lines. However, critical gaps of knowledge remain. Among them, the substrates and precise mechanisms of action of many auxiliary assembly factors remain poorly understood, especially those acting in early or intermediate assembly stages where pre‐ribosomal particles are unstable. Also, the mechanisms underlying tRNA^Val^ or tRNA^Phe^ incorporation into the mtLSU CP and the role of iron–sulfur cluster chaperones in delivering metal cofactors to coordinating pairs of proteins in the subunits await clarification. Additionally, the degradation pathways of misassembled ribosomes remain elusive. Moreover, the integration of mitoribosome assembly with mitochondrial and cellular processes such as mtDNA replication and transcription, Fe–S cluster biogenesis, and cellular response to energy status, nutrient availability, or stress remains to be fully characterized. Understanding these connections could aid in identifying targets for antibiotics that spare the mitoribosome, developing therapeutics for cancer treatment, or treating mitochondrial encephalomyopathies associated with organellar protein synthesis defects [5, 123].

## Conflict of interest

The authors declare no conflict of interest.

## Author contributions

All authors contributed to revising the literature, designing the outline of the article, and preparing drafts of the different sections. AB prepared the first full draft of the manuscript. MB, AS‐M, and AA prepared the figures. AB supervised the project and consolidated the style. All authors edited the manuscript and approved the final version.
